# Dietary fibre optimisation in support of global health

**DOI:** 10.1111/1751-7915.14542

**Published:** 2024-08-03

**Authors:** Anouschka S. Ramsteijn, Petra Louis

**Affiliations:** ^1^ Rowett Institute, University of Aberdeen Aberdeen UK

## Abstract

The human gut microbiota influences its host via multiple molecular pathways, including immune system interactions, the provision of nutrients and regulation of host physiology. Dietary fibre plays a crucial role in maintaining a healthy microbiota as its primary nutrient and energy source. Industrialisation has led to a massive decrease of habitual fibre intake in recent times, and fibre intakes across the world are below the national recommendations. This goes hand in hand with other factors in industrialised societies that may negatively affect the gut microbiota, such as medication and increased hygiene. Non‐communicable diseases are on the rise in urbanised societies and the optimisation of dietary fibre intake can help to improve global health and prevent disease. Early life interventions shape the developing microbiota to counteract malnutrition, both in the context of industrialised nations with an overabundance of cheap, highly processed foods, as well as in Low‐ and Middle‐Income Countries (LMICs). Adequate fibre intake should, however, be maintained across the life course to promote health. Here we will discuss the current state of dietary fibre research in the global context and consider different intervention approaches.

## Graphical abstract

Dietary fibre is an essential component of healthy diets. It exerts its actions on the human host largely via the intestinal microbiota. The interplay of fibre, microbiota and health are discussed in the context of different lifestyle settings across the globe. Finally, fibre‐based intervention strategies to improve health are considered.

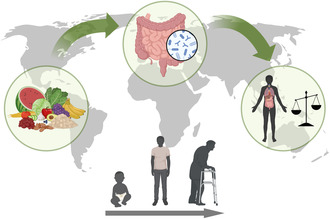

## INTRODUCTION

A suboptimal diet is thought to play a major role in global morbidity and death, with the leading dietary risk factors including insufficient whole grain, fruit, nut, seed and vegetable intakes. Those ingredients are important contributors to dietary fibre, whose deficiency was also identified as a large contributor to morbidity and mortality (Afshin et al., 2019). Dietary fibre consists of predominantly plant‐based carbohydrates that are indigestible in the upper human intestine and constitute a major nutrient and energy source for the complex microbial community in the large intestine, the colonic microbiota. Alarmingly, not a single geographic region across the world achieves the minimum fibre intake estimated to be required for optimal health (24 g/day for adults, Afshin et al., 2019).

**FIGURE 1:**
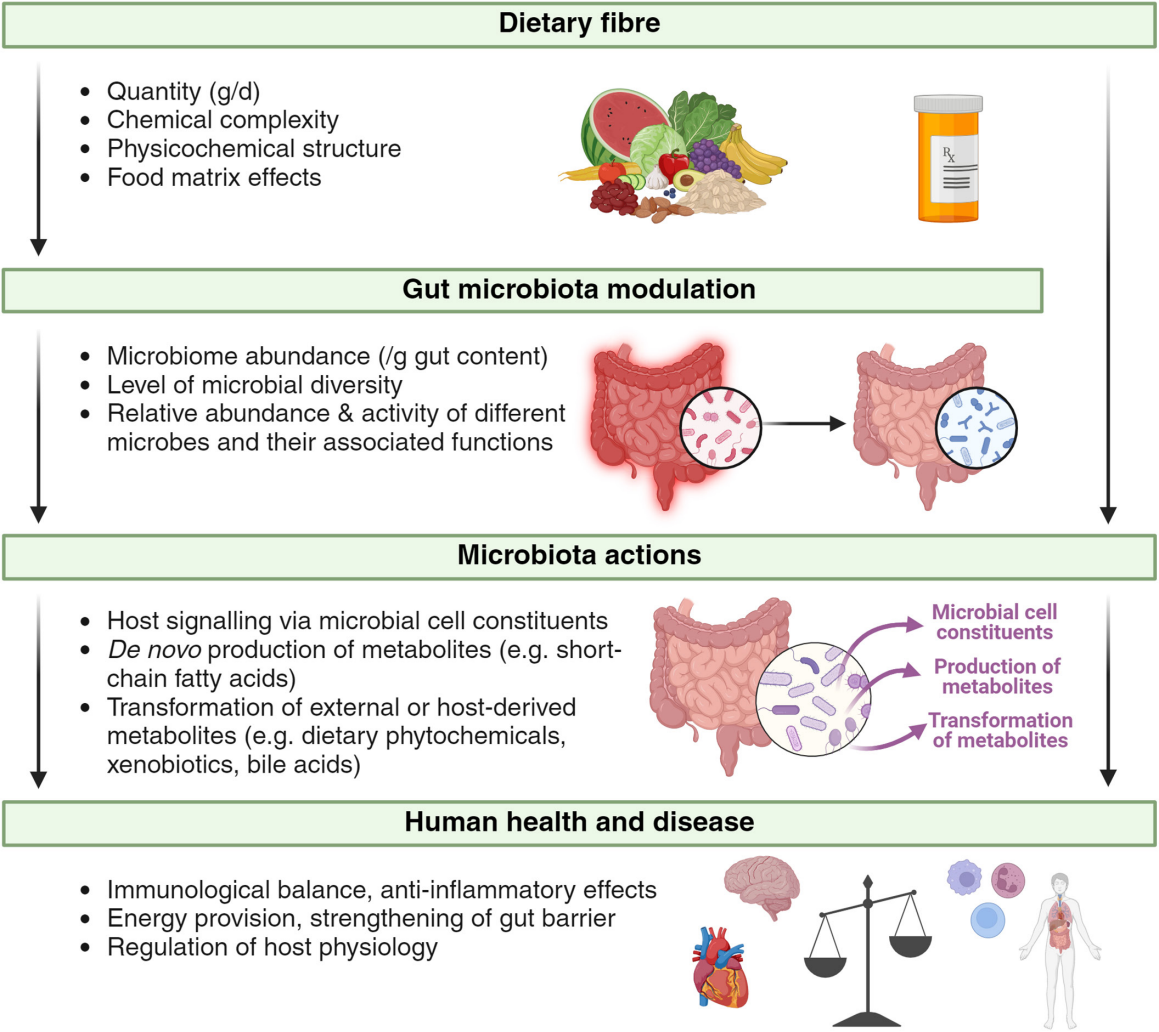
The impact of dietary fibre on the human gut microbiota and health.

Insufficient fibre intake has been associated with several non‐communicable diseases, including metabolic disorders, inflammatory diseases and cancer (O'Grady et al., 2019). Many of the health‐protective effects of fibre are due to the actions of gut microbes, which interact with the host either via their cell constituents (for example microbial cell envelope components) or via their metabolic activities (Figure 1). The microbiota ferments dietary fibre to several metabolites, including the short‐chain fatty acids (SCFAs) acetate, propionate and butyrate, known to exert health‐promoting effects on the host (de Vos et al., 2022). In addition, gut microbes are involved in the metabolism of numerous compounds present in the gut, including exogenous molecules such as dietary plant secondary compounds and drugs, and host‐derived molecules such as bile acids. Metabolites can be an energy source for the host (particularly SCFAs), but can also exert regulatory functions, for example by interacting with receptors that trigger a host response (de Vos et al., 2022). As individual microbes differ in their metabolic capabilities, the overall composition of the gut microbiota and metabolome are dependent on which microbes are promoted by the type of dietary fibre consumed by the host (Louis et al., 2021). Therefore, dietary fibre interventions that aim to optimise the gut microbiota are an attractive target to improve global health and reduce disease.

Here we discuss the role of dietary fibre in shaping the human microbiota and potential health consequences at the early and late stages of life in different populations across the globe. We then focus on the adult gut microbiota, before considering fibre‐based intervention strategies to improve human health in different settings.

## FIBRE AND THE GUT MICROBIOTA ACROSS THE LIFE COURSE

**FIGURE 2:**
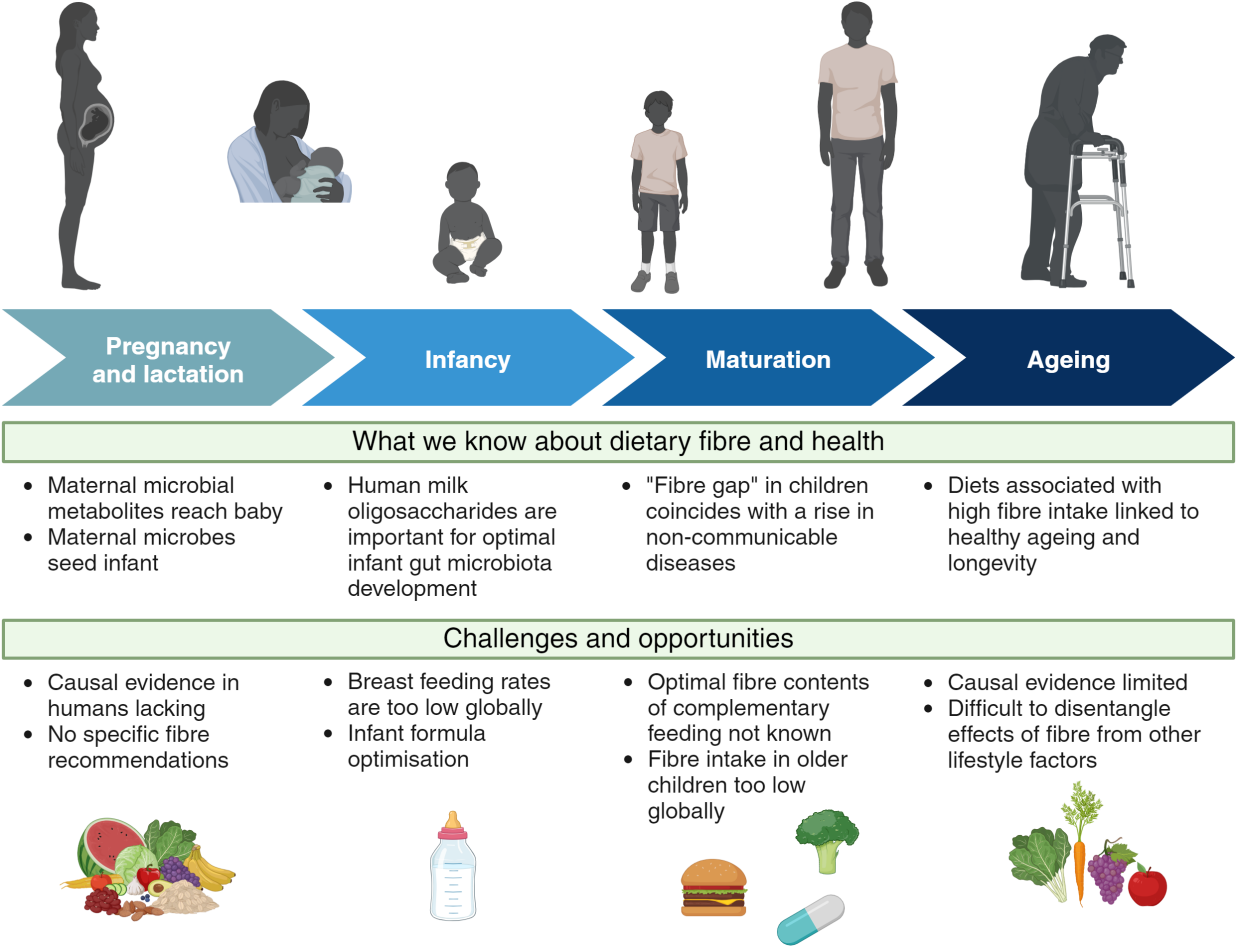
Dietary fibre across the life course: Opportunities and challenges.

During early life, maternal dietary fibre intake may contribute to offspring health and development by increasing the availability of fibre‐derived microbial products transferred through the placenta and breast milk (Figure 2). For instance, in rural Zimbabwe, the composition of the maternal microbiota was associated with birth weight and early life growth in infants (Gough et al., 2021). The abundance of specific resistant starch‐degrading taxa in the maternal gut, along with measures of gut microbiota diversity, were important predictors of neonatal growth. However, causality was not established, and the effects could reflect the extent of dietary deprivation, which likely affects child growth through microbiota‐dependent and ‐independent pathways. Preclinical experiments provide the first causal evidence linking maternal dietary fibre intake to biomarkers of offspring health. For example, a maternal fibre‐free diet in mice colonised with a consortium of 14 microbes altered gene expression and immune cell populations in the pups' colons during weaning (Grant et al., 2023). In this study, maternal fibre‐poor versus fibre‐rich diet also modulated pup microbiome assembly. Therefore, both maternal and pup microbial activity may mediate the effect of dietary fibre on early‐life immune system development. Indeed, in humans, postnatal mother‐infant transmission of microbes supports healthy immune functioning (Browne et al., 2022). Suboptimal microbial transfer, as in caesarean section versus vaginal births and with antibiotic use, is associated with vulnerability to infection with opportunistic pathogens and immune‐related illness in children. Enhancing mother‐infant microbial transmission could therefore optimise the effects of maternal dietary fibre intake on child health, as beneficial maternal microbes promoted by fibre may shape the infant's initial microbiome assembly.

In terms of infant nutrition, exclusive breastfeeding for 6 months is recommended by the World Health Organization (WHO). Human milk contains a highly complex and individualised mixture of nutrients and bioactive molecules, including human milk oligosaccharides (HMOs), that evolves over the course of lactation. HMOs, like dietary fibre, cannot be digested by the host but are utilised by microbes in the infant gut, promoting the growth of a specific set of beneficial microbes such as bifidobacteria, which support gut immune barrier development (Kong et al., 2020). Infancy may therefore be the only life stage at which a relatively low gut microbial diversity is a sign of health. In light of the crucially important role of HMOs (and in addition to the variety of other beneficial components of breast milk), it is worrying that less than half of global infants are breastfed the recommended amount of time, caused by a complex interplay of factors that differ across countries and populations, including individual (e.g. low breast milk production or infant irritability), environmental (e.g. healthcare characteristics and workplace support) and structural factors (e.g. policy decisions in support of breastfeeding) (Perez‐Escamilla et al., 2023). Some infant formulas contain microbiota‐directed ingredients, or prebiotics, in an attempt to mimic the effects of HMOs, such as 2′‐fucosyllactose, galacto‐oligosaccharides, fructo‐oligosaccharides, and pectins. More research, development and testing of such ingredients is necessary to offer optimal nutrition to those infants who are not breastfed, specific to age and individual needs, in support of establishing a healthy gut microbiome with life‐long benefits (Kong et al., 2020).

After exclusive milk feeding, children are introduced to complementary foods, associated with dramatic changes in gut microbiota diversity and composition (Olm et al., 2022). These foods, usually soft, cooked, or pureed, may also be pre‐chewed by caregivers, a practice historically common and still prevalent, particularly in the Global South. Some advocate for its potential to prevent undernutrition (Pelto et al., 2010). Exploring the differences in dietary fibre content and diversity between premasticated foods and modern infant foods, as well as the timing of their introduction, could offer valuable insights. While the WHO does not specify dietary fibre recommendations for infants, concerns sometimes arise regarding high‐fibre content in complementary feeding, potentially impacting energy density and growth (Lutter et al., 2021). The balance between this risk and the risk of suboptimal gut microbiota development due to fibre deficiency likely depends on various factors, such as environmental context, intrinsic factors like susceptibility to under‐ or overweight, and exposure to infections. Context and diet significantly influence the trajectory and outcome of gut microbiota maturation, as evidenced by a meta‐analysis across 18 industrialised, transitional and non‐industrialised populations (Olm et al., 2022). Industrialisation correlates with a loss of specific microbial species in the infant's gut, possibly linked to rising childhood diseases in industrialised nations like impaired glucose regulation, obesity and inflammatory disorders. Furthermore, some gut microbial metabolites and microbiome maturation in infancy have been associated with protection against conditions like childhood asthma (Depner et al., 2020). Recommendations for children's dietary fibre intake vary by country and are largely based on adult guidelines, focusing on intake rather than quality or source, pending further research (Hojsak et al., 2022). In the UK, targets range from 15 g/day for ages 2–5 to 30 g daily for ages 16–18 but mean fibre intake in children falls below these levels across all age groups.

Apart from its role during early life and maturation, dietary fibre has gained increased attention due to its purported contribution to healthy ageing. Diets that have been associated with health later in life, such as the Mediterranean diet and dietary patterns in “Blue Zones”, where populations display exceptional longevity, generally contain a relatively high intake of dietary fibre. In addition, the composition of the gut microbiome in older individuals has been suggested to be linked to frailty and cognitive functioning and has been proposed as a target to extend healthspan (Ticinesi et al., 2024). Whether there is a causal link between dietary fibre intake and healthy ageing remains to be established, given that there are a variety of other contributing factors, like social‐ and psychological health, physical activity and genetic background, that could be (partially) responsible for the effects observed (Pes et al., 2022). Randomised controlled trials with dietary interventions are warranted.

## THE GUT MICROBIOTA, FIBRE AND HEALTH IN DIFFERENT POPULATIONS

Culture‐independent microbiota analyses initially focussed on individuals living in industrialised societies, which resulted in attempts to define a core microbiome associated with health and microbiome signatures associated with disease. However, many different factors determine microbial colonisation, leading to large inter‐individual variation. Host genetics has a relatively minor influence, whereas diet and lifestyle factors have a major impact on gut microbiota composition (Parizadeh & Arrieta, 2023). Furthermore, different microbes may have a similar effect on aspects of host health in different individuals due to microbial functional redundancy. There is therefore no single ‘healthy microbiome’, and associations found with certain disease states to date usually lack the establishment of causality and often are not reproducible across studies (Brüssow, 2020). High microbial diversity in adulthood has, however, repeatedly been found to be associated with health, which may go hand in hand with a more diverse ecosystem being more resilient to perturbation (McBurney et al., 2019). Furthermore, based on our understanding of the activities that different microbes exhibit, we can make some inferences on whether a microbe is likely to promote health. Both potentially beneficial and detrimental traits may be present within the same microbe and traits can vary even between closely related microbes. For example, *Faecalibacterium duncaniae* (formerly *Faecalibacterium prausnitzii*) strain A2‐165 produces the anti‐carcinogenic and anti‐inflammatory metabolite butyrate, but was also shown to exhibit exceptionally high β‐glucuronidase activity in vitro, which is associated with an increased risk of colorectal cancer (McIntosh et al., 2012). In addition, host exposures and lifestyle factors also need to be taken into consideration to estimate the health impact of certain microbial traits. For example, while β‐glucuronidase activity has been implicated in disease via its action on numerous carcinogens and drugs, it may also promote health via liberation of dietary secondary plant compounds (Gao et al., 2022).

As lifestyle, diet and environmental exposures to xenobiotics vary widely in different societies and populations across the globe, our view of what constitutes a ‘normal’ microbiota based on industrialised societies is too narrow, and recent studies have revealed profound microbiome differences between different populations (Shanahan et al., 2022; Table 1). From an evolutionary perspective, early humans adapted to a hunting lifestyle, however, plants (in particular roots and tubers) likely remained an important nutritional component. Based on modern hunter‐gatherer societies, daily dietary fibre intakes may have been in the order of 70–130 g, much higher than the current targets of around 20–40 g in industrialised societies (Tannock, 2024). A study targeted at cellulose‐degrading ruminococci revealed that they are more prevalent and abundant in humans in non‐industrialised societies, in agreement with their consumption of cellulose‐rich diets (Moraïs et al., 2024, Table 1). Higher abundance of *Treponema* species has also been found in traditional societies, a genus known to harbour pathogenic species (for example *T. pallidum*, *T. denticola*). The species found in traditional societies, however, are most closely related to a carbohydrate‐utilising swine commensal, *T. succinifaciens*, and may be true commensals contributing to fibre breakdown (Obregon‐Tito et al., 2015, Table 1). Ultra‐deep sequencing of Hadza hunter‐gatherers from Tanzania has identified many new microbial species and allowed for an in‐depth analysis of taxa associated with industrialised populations, and taxa that are under‐represented or lost relative to traditional societies (Carter et al., 2023). Analysis of the functional capacity encoded in the sequenced genomes also revealed differences (Table 1), which is broadly in agreement with a recent faecal metabolome study across several societies that demonstrated differences across an industrialisation gradient for some metabolites (for example amino acid‐conjugated bile acids, Haffner et al., 2022).

**TABLE 1:** Examples of recent studies and approaches used to evaluate microbiome differences in traditional and industrialised populations.

Study populations and experimental approach	Main outcomes	Reference
Matses hunter‐gatherers (Peruvian Amazon), Tunapuco traditional agriculturalists (Andean highlands), Norman residents (Oklahoma) 16S rRNA amplicon and metagenomic sequencing	Traditional lifestyle: higher microbial richness, higher *Succinivibrio*, *Treponema*, *Prevotella* *Treponema* in traditional societies diverse, related to *T. succinifaciens* Industrial lifestyle: higher *Bifidobacterium, Bacteroides* Complex differences between populations in Firmicutes species	Obregon‐Tito et al. (2015)
Hadza hunter‐gatherers (Tanzania), Nepalese foragers and agrarians, Californians Ultra‐deep metagenomic sequencing	Higher bacterial and archaeal diversity in Hadza, recovery of many new species and genes 124 species vanishing in industrialised populations Spirochaetota species decrease with increased industrialisation California: enriched in oxidative stress genes (adaptation to inflammatory processes?)	Carter et al. (2023)
Identification of key ruminococcal cellulosome genes in metagenome‐assembled genomes of human and rumen origin	Identification of new *Ruminococcus* species with higher abundance and prevalence in ancient human, hunter‐gatherer and non‐westernised societies	Moraïs et al. (2024)
Three rural lifestyles across a hunter‐gatherer ‐ agrarian gradient: forest‐dwelling Baka, Baka villagers, Nzime farmers (Cameroon); integration with available data from worldwide populations Metagenomic sequencing	Lifestyle‐associated abundance differences of several species and genes Identification of species and genes likely involved in wild plant consumption based on functional screening of genome‐scale metabolic networks Identification of secondary metabolite biosynthetic gene clusters specific to rural individuals	Rampelli et al. (2024)

The onset of farming resulted in a major diet and lifestyle shift. While grass seeds are believed to have been part of the hunter‐gatherer diet, grains (especially wheat and rice) became major staple foods, alongside other crops and farmed animals (Tannock, 2024). Grains are Monocotyledon plants with large cell wall compositional differences to Dicotyledon fruits and vegetables (Louis et al., 2021), consequently promoting the growth of different gut bacteria (Solvang et al., 2023). The picture of microbiome differences between different contemporary lifestyles (hunter‐gatherer, traditional agrarian, industrialised, rural/urban, etc.) is still emerging. However, together with evidence from microbiome changes of migrants associated with lifestyle changes and diet swap studies (O'Keefe et al., 2015; Shanahan et al., 2022), there is no doubt that diet and lifestyle have a major impact on the microbiome, with potential knock‐on effects on host health. Accordingly, it was recently suggested that microbial extinctions during industrialisation within the quickly evolving gut microbiota may result in a mismatch with the more slowly evolving human genome in industrialised societies. This may lead to misregulation between microbe and host aspects of the human holobiont and the development of non‐communicable chronic diseases, which are on the rise in industrialised nations (Sonnenburg & Sonnenburg, 2019). The optimisation of dietary fibre intake is therefore a promising target for health improvement across a wide range of societal settings, by developing tailored, affordable and practicable interventions.

## DIETARY FIBRE OPTIMISATION STRATEGIES

**FIGURE 3:**
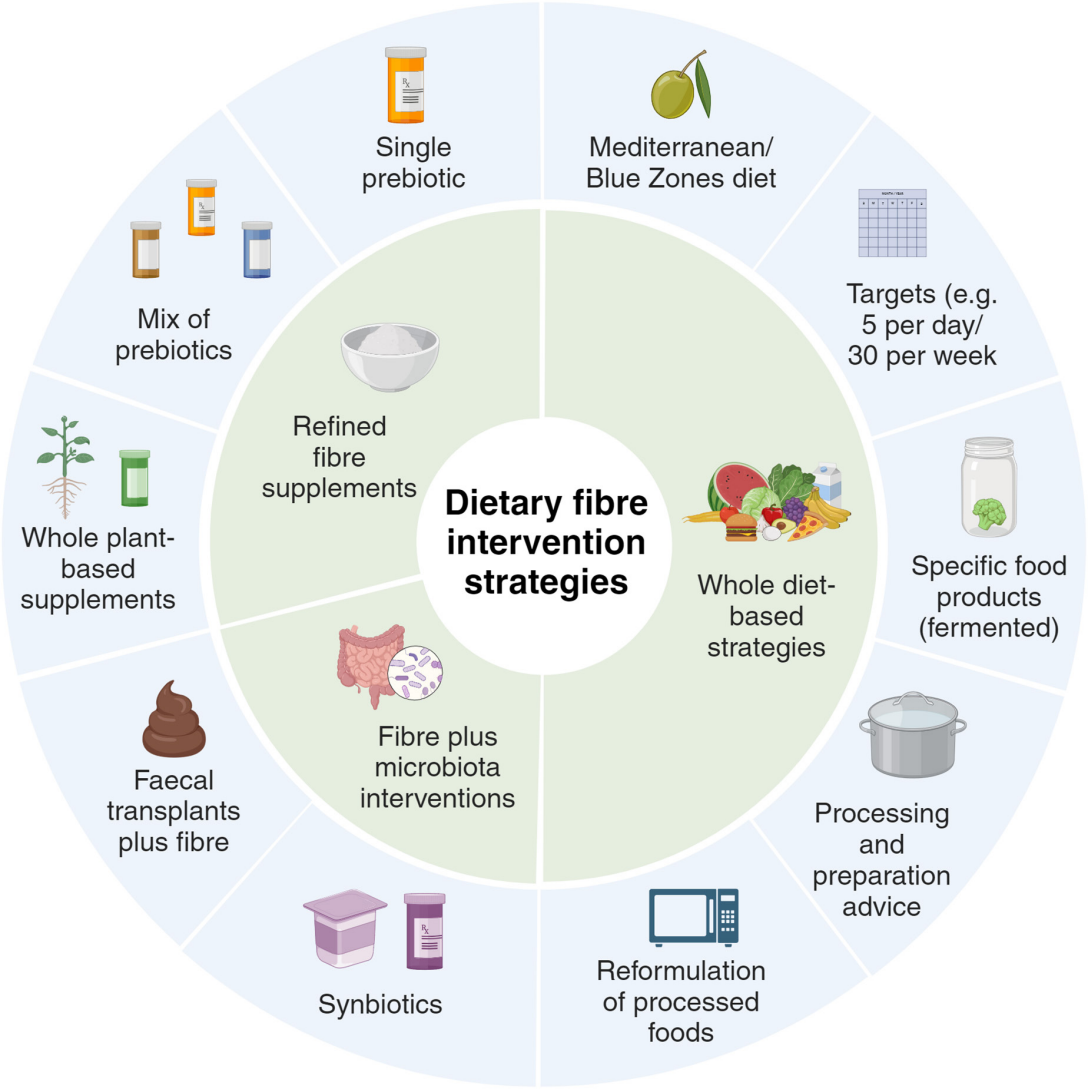
Dietary fibre intervention strategies.

The recent trend towards ultra‐processed foods at the expense of whole plant‐based diets is in contravention with dietary guidelines across the world and increases chronic disease risk via several mechanistic routes involving the microbiota (Armet et al., 2022). Improved fibre intake may also provide protection against infection with pathogenic microbes or gastrointestinal parasites by strengthening of the gut barrier function of the commensal microbiota (colonisation resistance) and by supporting immune functioning. This could have important health implications, for instance in the context of malnutrition and growth stunting (Gabain et al., 2023). However, the quality and quantity of fibre leading to optimal health outcomes will depend on the target population and, at the level of the individual, specific physiological and microbiome traits may also have to be taken into consideration (Armet et al., 2022; Simon et al., 2023; Figure 3). In general, there is mounting evidence that current fibre recommendations may not be sufficient and that we need to aim for higher intakes (>50 g/day) to achieve optimal health outcomes (O'Keefe, 2019).

Dietary fibre ingredients in the form of purified individual non‐digestible carbohydrates (prebiotics such as fructans) are available, and while they are easy to incorporate into food products, they may not be ideal as major fibre intervention tools (Louis et al., 2016). High doses of single purified carbohydrates are unlikely to promote a diverse microbiota, and a more complex mix of fibre types may be required to achieve significant health benefits across a range of lifestyles. What effect the physicochemical properties of fibre have on the microbiota is not well understood (Puhlmann & de Vos, 2022), but it is to be expected that eating a diverse whole plant‐based diet is more beneficial than even a mixture of several refined fibre components. The insoluble plant particles provide surfaces for biofilm formation, which could foster interactions between different microbes, for example, synergistic actions in complex fibre breakdown or cross‐feeding of breakdown intermediates or fermentation products between microbes (Solvang et al., 2023). Complex insoluble fibre is also likely to be more slowly fermented, leading to more sustained microbial activity and production of health‐promoting metabolites further into the distal gut, where most gut diseases occur. Dietary interventions targeting sustained improvements in whole plant‐based food intakes are therefore desired, but other approaches also need to be implemented to achieve meaningful improvements in fibre intakes across different populations.

In Low‐ and Middle‐Income Countries (LMICs) in particular, any interventions focused on whole diet‐based strategies will have to consider local food availability and affordability as well as cultural practices. Interventions with purified pre‐ and synbiotics (combinations of prebiotics and probiotic microbes) have recently been successfully implemented in low‐resource contexts because of their affordability, acceptability, and scalability (Momo Kadia et al., 2023). They were specifically aimed at improving resilience against infection and promoting growth in early life in LMIC populations at risk of malnutrition and stunting and are a safe and practical means of improving population health at a critical and vulnerable developmental stage. Other strategies to combat malnutrition include supplemental foods that are locally available and acceptable, targeting the microbiota. In a randomised controlled trial of a microbiota‐directed complementary food versus an existing standard complementary food, children with moderate acute malnutrition recovered better on the microbiota‐directed food, even though it contained fewer calories (Chen et al., 2021). Paradoxically, dietary fibre interventions likely have benefits in the context of both over‐ and undernutrition by promoting a healthy and balanced gut microbiota.

In industrial societies with ready access to cheap, processed and highly palatable foods, it can be difficult to change behaviour towards more whole plant‐based diets. Novel complex fibre food ingredients therefore need to be developed alongside the more traditional prebiotics to aid the reformulation of processed foods with the aim of improving their healthiness. Other factors, including sustainability, environmental impact, climate change, food production and distribution, cost‐effectiveness, intervention effectiveness (at consumer and policy level, for example, implementation of fibre‐rich foods in schools and hospitals) and local conditions, also need to be taken into consideration (Afshin et al., 2019; Jennings et al., 2024).

For individuals in industrialised societies who have lost crucial microbes for dietary fibre breakdown, an increased fibre intake may have to go hand in hand with replenishing the gut with fibre degraders to unlock the full fibre potential. On the flip side, adequate fibre intakes must be sustained to achieve long‐term improvements in gut microbiota composition and health outcomes. Whether individual strains (next‐generation probiotics) or strain cocktails are sufficient to establish lost microbes in the gut, or if more drastic interventions such as faecal transplantation are advisable, remains to be established. Finally, the emerging field of personalised nutrition holds promise to promote health or target specific diseases via optimisation of the diet, by taking host genetics and/or microbiome makeup of the individual into account (Simon et al., 2023).

## CONCLUDING REMARKS

The microbiome field is transitioning from mainly characterisation‐based research to investigating the mechanisms of its functioning as a microbial ecosystem and its interactions with diet and the human host. Together with the acceleration in our understanding of host physiology, largely driven by technological advances and the exploitation of the Human Genome Project, and exciting developments in nutrition research, we are currently in an ideal position to make a major impact on global health by developing dietary strategies targeting the microbiome. To achieve this, we need tailored strategies addressing the diverse requirements of different human populations and considering the entire food system. Practical and effective solutions require a concerted effort by academia, industry, governments, and non‐governmental organisations. Crucially, in addition to improving dietary fibre intakes in societies with either insufficient or excess food supply, we must also focus on ensuring smooth transitions for populations that are moving from traditional to industrialised lifestyles, avoiding the fibre‐poor Westernised diet stage. Humans have evolved to crave foods high in sugar and fat, which is beneficial when food is scarce, but is a problem in industrialised societies. However, some individuals genuinely enjoy mostly unprocessed, whole plant‐based diets and such dietary habits should be encouraged. This strategy may be particularly successful in early childhood when food preferences are formed. But even for adults, it is relatively easy to modify taste preferences such as sweetness over a short period of time. However, to achieve meaningful improvement in fibre intake across entire populations, we will also require food reformulation strategies to make modern food products healthier. Additionally, making options with higher (and more diverse) dietary fibre contents affordable, accessible, palatable, and easy to prepare is crucial for supporting the long‐term health, wellbeing, and economic potential of individuals and societies.

## AUTHOR CONTRIBUTIONS

**Anouschka S. Ramsteijn:** Conceptualization; writing – original draft; writing – review and editing; visualization. **Petra Louis:** Conceptualization; writing – original draft; writing – review and editing; visualization.

## CONFLICT OF INTEREST STATEMENT

The authors declare no conflict of interest.
